# Multimodal artificial intelligence models for radiology

**DOI:** 10.1093/bjrai/ubae017

**Published:** 2025-01-17

**Authors:** Amara Tariq, Imon Banerjee, Hari Trivedi, Judy Gichoya

**Affiliations:** Mayo Clinic, Phoenix, AZ, 85054, United States; Mayo Clinic, Phoenix, AZ, 85054, United States; Department of Radiology and Imaging Sciences, Emory University, Atlanta, GA 30322, United States; Department of Radiology and Imaging Sciences, Emory University, Atlanta, GA 30322, United States

**Keywords:** multimodal AI, AI in radiology, vision-language models for radiology

## Abstract

Artificial intelligence (AI) models in medicine often fall short in real-world deployment due to inability to incorporate multiple data modalities in their decision-making process as clinicians do. Clinicians integrate evidence and signals from multiple data sources like radiology images, patient clinical status as recorded in electronic health records, consultations from fellow providers, and even subtle clues using the appearance of a patient, when making decisions about diagnosis or treatment. To bridge this gap, significant research effort has focused on building fusion models capable of harnessing multi-modal data for advanced decision making. We present a broad overview of the landscape of research in multimodal AI for radiology covering a wide variety of approaches from traditional fusion modelling to modern vision-language models. We provide analysis of comparative merits and drawbacks of each approach to assist future research and highlight ethical consideration in developing multimodal AI. In practice, the quality and quantity of available training data, availability of computational resources, and clinical application dictates which fusion method may be most suitable.

## Introduction

Multimodal data analysis is a routine part of clinical decision making. Clinicians often review multiple streams of heterogenous data elements including imaging, labs, clinical notes, physical examination findings, medications, and comorbidities to make diagnostic or prognostic decisions. Early efforts in deep learning models in healthcare typically focused on single modalities—for example, imaging, labs, or a finite set of features from the electronic health record (EHR). Most radiology models today are developed using convolutional neural networks (CNNs) applied to a single image type like chest X-rays or head CTs.^1^^,^^2^ Even basic elements like patient demographics are rarely included in the model pipeline. While image-only models can perform well on image-only tasks such as haemorrhage detection, there remains a fundamental gap for artificial intelligence (AI) models that operate on imaging alone to outperform physicians in clinical decision making as they don’t have the same information available. When models are developed without including pertinent clinical information, they may lead to non-specific or inappropriate conclusions that limit their utility. For example, a clinically deployed model for intracranial haemorrhage at our institution notifies radiologists multiple times daily about acute haemorrhages for in-patients that are postoperative, for whom intracranial blood products are expected thus unnecessarily disrupting the radiologist workflow. Additionally, we observed a 10% performance drop in outpatients in a pulmonary embolism triage model compared to the reported FDA validation metrics. This is concerning as the outpatients are the most vulnerable when they have critical findings as they cannot receive timely intervention. Building single modality models without clinical context (available from multimodal data) ultimately results in impractical models with limited clinical utility.

Advancements in computational methods have made it possible to merge clinical and imaging data when training models, as well as incorporate multiple imaging modalities into the same model (eg, combining mammograms with ultrasound and MRI for breast cancer screening^3^) These may even be combined with digital histopathology slides to predict treatment response and post-diagnosis prognostication.^4–6^ The interest in multi-modal fusion has resulted in many targeted funding opportunities and release of many multimodal large datasets like INSPECT,^7^ ROCO,^8^ All of Us,^9^ and UK Biobank.^10^ Many new multi-modal fusion modelling techniques have also been developed that combine radiology images along with clinical information from the EHR to make diagnoses or predictions.^11–15^ This survey paper reviews various computational approaches that can be applied to multimodal datasets in radiology based on model architecture and discusses them from technical and application perspectives including relevant ethical concerns.

### Traditional fusion models

**Figure 1.**
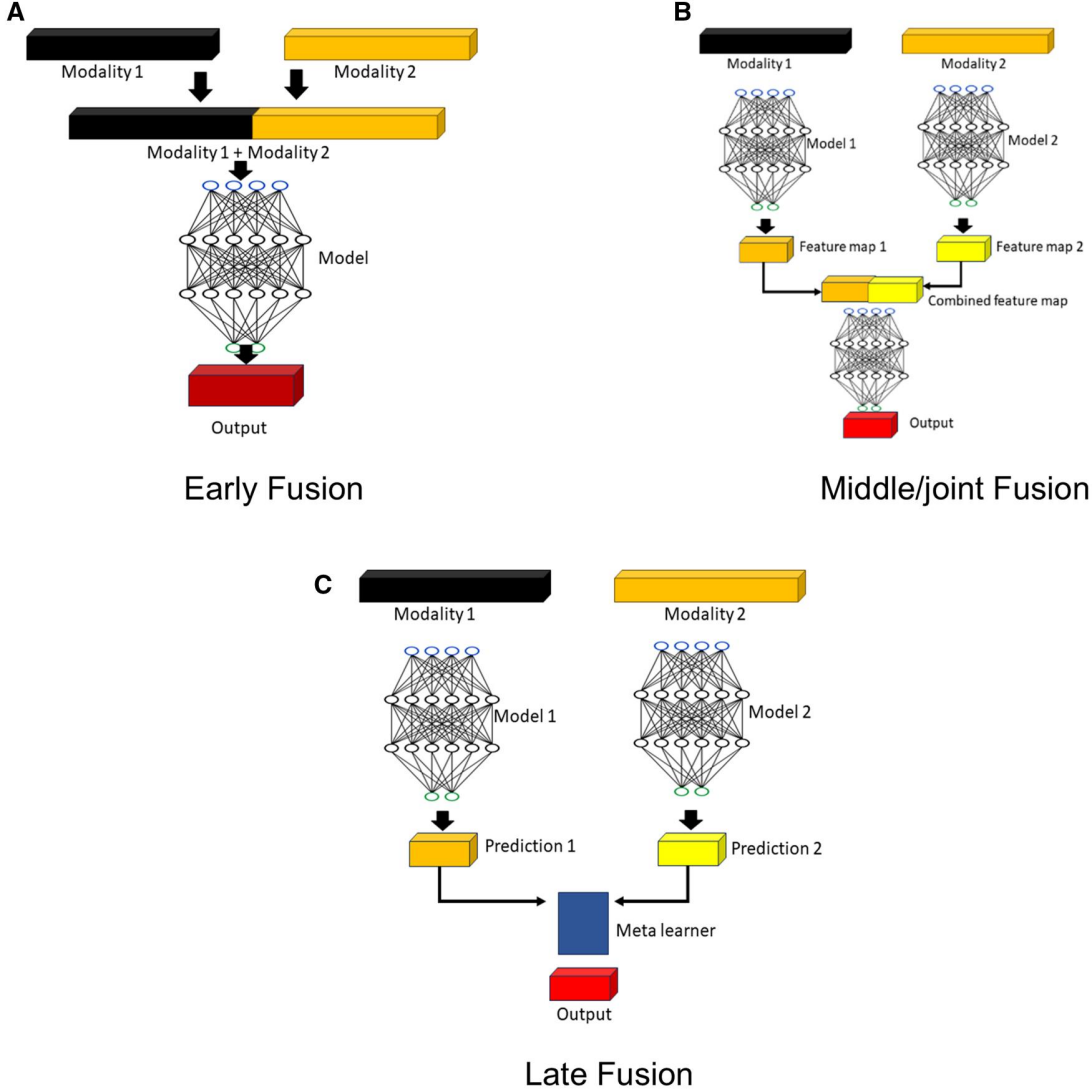
Traditional fusion models—(A) early fusion, (B) joint/middle fusion, and (C) late fusion. These architectures differ based on the point of merging multiple features early, middle, or late in the model pipeline.

The earliest form of fusion models in machine learning combined tabular data from multiple sources (eg, lab test results, demographics), transformed or harmonized them, and trained an end-to-end model. These models are straightforward in terms of implementation and are explainable in terms of model-assigned importance to input features. However, introduction of complex, unstructured, and high dimensional data modalities like images and free text has motivated advanced research that combines straightforward fusion frameworks with complex pretrained feature extraction pipelines (Figure 1).^11^

*Late fusion* is a relatively simple framework in which scores/probabilities generated by different models are parsed via a meta-learner to calculate a weighted combination of the decision as summarized in Figure 1C. Each model is trained in isolation on different modalities and since only target probabilities are “fused,” no architectural innovation is needed to fuse different modalities like images and tabular data. However, this technique is limited in that the fusion model cannot learn complementary information from different modalities as the features or probabilities are already extracted and frozen from each modality *before* fusion. Although limited compared to more advanced methods, many models have successfully used this approach to combine radiology images like chest X-rays with clinical data, achieving greater performance compared to individual modality models.^16^^,^^17^

In contrast, *Early fusion* strategies fuse raw data or extracted features (often by straightforward concatenation) early in model training and then pass the combined features through a model with loss propagated back up to the fusion layer.^11^ While this approach allows the model to extract complementary information from 2 or more modalities, the heterogeneous nature of data, for example, images and tabular EHRs typically requires features to be extracted from unstructured data (images and text) instead of raw data before they can be fused. This suggests that the quality of the pretrained feature extractor can significantly influence the performance of the final classification model, highlighting the necessity of careful selection of the feature extractor, and potential need to experiment with multiple variations of feature extraction. For example, ImageNet-trained CNN models are not ideal feature extractors for chest X-rays due to differences between natural images (ImageNet dataset) and chest X-rays. Instead features extracted from pipelines trained or fine-tuned on medical domain images have demonstrated better model performance.^18^

Finally, *joint fusion* combines components of early and late fusion and allows end-to-end training of models (ie, backpropagation of loss to the input layer), however, with independent parallel feature extractors that derive relevant features from each modality that are then combined using linear or nonlinear functions.^11^^,^^12^ In joint fusion, the feature extraction backbone can be updated to better suit the downstream prediction task.

A limitation of all traditional fusion approaches is the inability to handle missing data, i.e., all modalities must be available during both training and inference. Early and joint fusion models may also overfit due to the high dimensionality of the fused feature sets and often underperform compared to the baseline single modality models. In addition, developers use feature selection methods and manual curation based on domain knowledge to overcome limitations of joint learning opportunity in late fusion models, however, this can limit widespread deployment of developed models where the same curation techniques may not apply. These limitations have led to the development of innovative fusion methods with capacity to integrate both explicit and implicit information with minimal human curation.

### Graph-based fusion models

While traditional fusion paradigms result in rigid models with restrictions on features that can be processed, graph convolutional neural networks (GCNs) provide an opportunity to fuse implicit information about clinical similarity between samples or patients with information extracted from multiple clinical modalities like images, text, or tabular data. This architecture has proven to be effective for several tasks where instances can form meaningful and informative “neighbourhoods.”^14^^,^^19–22^ For example, information on Alzheimer disease progression is present both in brain MRIs and in the demographics and clinical history of a patient.^22^ Instead of fusing demographic information directly with features extracted from brain images under an early or late fusion paradigm, graph-based models can learn the relationship between brain images of a specific patient (node feature vector) and brain images of other clinically similar patients (feature vectors of other connected nodes) as defined by neighbourhoods in the graph structure that connects patients with similar demographic characteristics (edge feature vectors). This approach has proven its merit in several use cases including diagnosis and clinical event prediction.^14^

**Figure 2.**
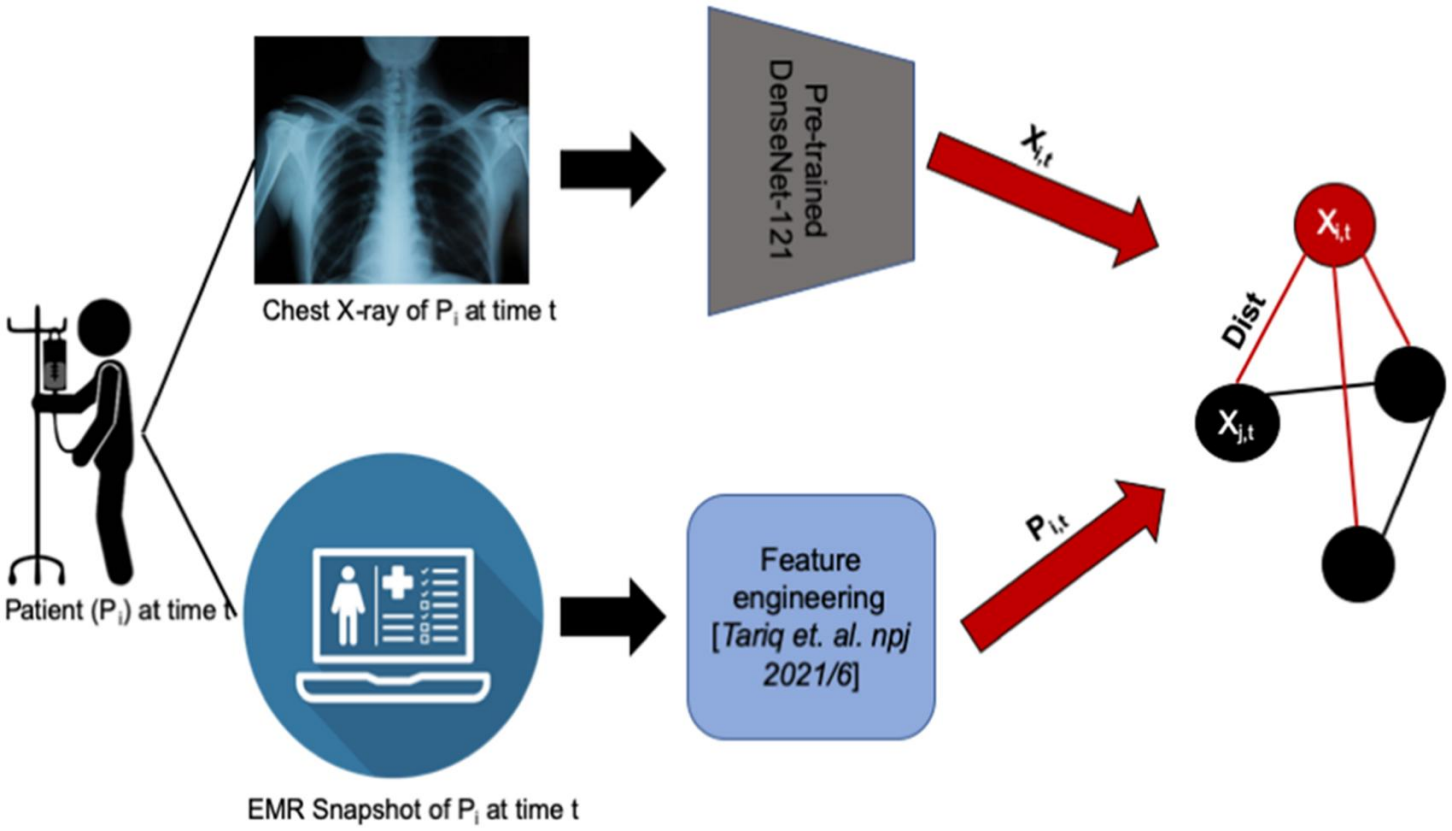
Graph convolutional neural network adaptation to predict COVID outcomes using multimodal data—chest X-ray and tabular EHR. The image is adapted from Tariq et al.^8^

GCNs have been adapted for a wide range of modalities including textual, tabular, and imaging data, indicating their modality-agnostic benefits. They also demonstrate better generalizability capabilities for missing data, especially if the missing data was only used for graph structure formation.^23^^,^^24^ A trained GCN model expects a graph structure as input and does not place any restriction on the graph formation process. Hence, any missing features can be omitted from the graph formation process during external validation while leaving the pretrained GCN applicable. GCNs have also been modified to include temporal information by including temporal convolutional layers.^19^ Such models allow incorporation of time-varying clinical information, like laboratory values or vital signs (Figure 2). For example, a temporal model was used to build edges of the graph such that patients (nodes) with similarly evolving clinical status were connected and used to predict the risk of blood transfusion for each patient.^23^ To demonstrate the variable performance of fusion architectures, we evaluated GCN and traditional fusion for 2 different tasks; (a) Discharge from hospital and mortality prediction for COVID-19 patients by fusing chest X-rays and procedure and billing codes; (b) transfusion risk prediction for in-patients by fusing acquired features (laboratory test results and vital signs) and derived clinical features (billing and procedure codes and prescribed medications) [17]. These experiments were externally validated in addition to evaluation on internal held-out test sets. For the first task, models were trained using cohort of Emory University Hospital patients and externally validated on a cohort from Mayo Clinic. For the second task, the model was trained using patient cohort from Mayo Clinic and externally validated on the publicly available MIMIC-III dataset – see result summary in Table 1.

**Table 1.** Performance comparison between GCN and traditional fusion approach of late fusion across 2 different use cases—COVID-19 discharge and mortality prediction; and blood transfusion risk.

Model	Internal			External		
	Sensitivity	Specificity	AUROC	Sensitivity	Specificity	AUROC
Hospital discharge prediction						
Late fusion	69.7 [68.4-70.8]	65.5 [64.1-67.2]	74.5 [73.4-75.6]	51.2 [50.4-51.9]	61.3 [60.0-62.7]	58.1 [57.2-59.0]
GCN	71.1 [69.9-72.3]	69.6 [68.2-71.4]	77.1 [76.1-78.2]	64.8 [64.1-65.4]	57.4 [55.9-58.8]	64.6 [63.7-65.5]
	Mortality prediction					
Late fusion	85.6 [83.0-88.5]	81.1 [79.8-82.8]	88.6 [87.2-90.2]	76.0 [74.0-78.3]	82.2 [81.1-83.4]	81.6 [80.3-82.9]
GCN	84.7 [82.3-87.9]	82.5 [81.1-83.9]	90.1 [89.0-91.3]	81.4 [79.6-83.5]	74.6 [73.2-76.0]	85.3 [84.2-86.5]
	Transfusion risk prediction					
Late fusion	64.0 [62.1-66.0]	64.2 [62.9-65.5]	69.9 [68.6-71.2]	60.0 [50.0-75.0]	54.0 [50.8-57.9]	44.4 [31.3-56.0]
GCN	73.8 [72.0-75.6]	65.4 [64.1-66.7]	77.4 [76.3-78.5]	80.0 [66.0-95.0]	69.8 [66.7-73.6]	70.8 [62.5-84.7]

Although GCNs are powerful, unstructured modalities like images and text may still need pretrained feature extractors for generating node feature vectors. Careful selection of the feature extraction pipeline is still required for traditional fusion architecture. GCN architectures may also suffer from a “homogenization” effect if too many graph convolutional layers are included in the model. For example, imaging features extracted from pretrained CNN pipelines for different chest X-rays have similar characteristics as they share overall image structures such as a dark background and human-shaped foreground object. When used as node features, the first layer of graph convolution will process one patient’s chest X-ray (node feature vectors) along with chest X-rays of its directly connected neighbours, often through weighted combinations. A second layer will involve node feature vectors of *neighbours of neighbours* of the node. As additional graph convolution layers are added, the processed forms of all chest X-rays (node features) will become increasingly similar and the GCN will lose discriminatory ability for differences between the images (eg, disease states).

Model explainability is another challenge of graph-based models. While feature weights can be used as explanations in early or late fusion models, GCN-based models require 2 forms of explanation—explanation of important node features and importance of graph neighbourhood or subgraphs. Explanatory frameworks like GNNExplainer have been proposed which randomly sample graph neighbourhoods and estimate their effect on model output, thus assigning importance weights to graph edges.^15^^,^^25^ However, clinical applicability of such explanations is still under-explored due to their complexity.

### Joint embedding of multimodal data

**Figure 3.**
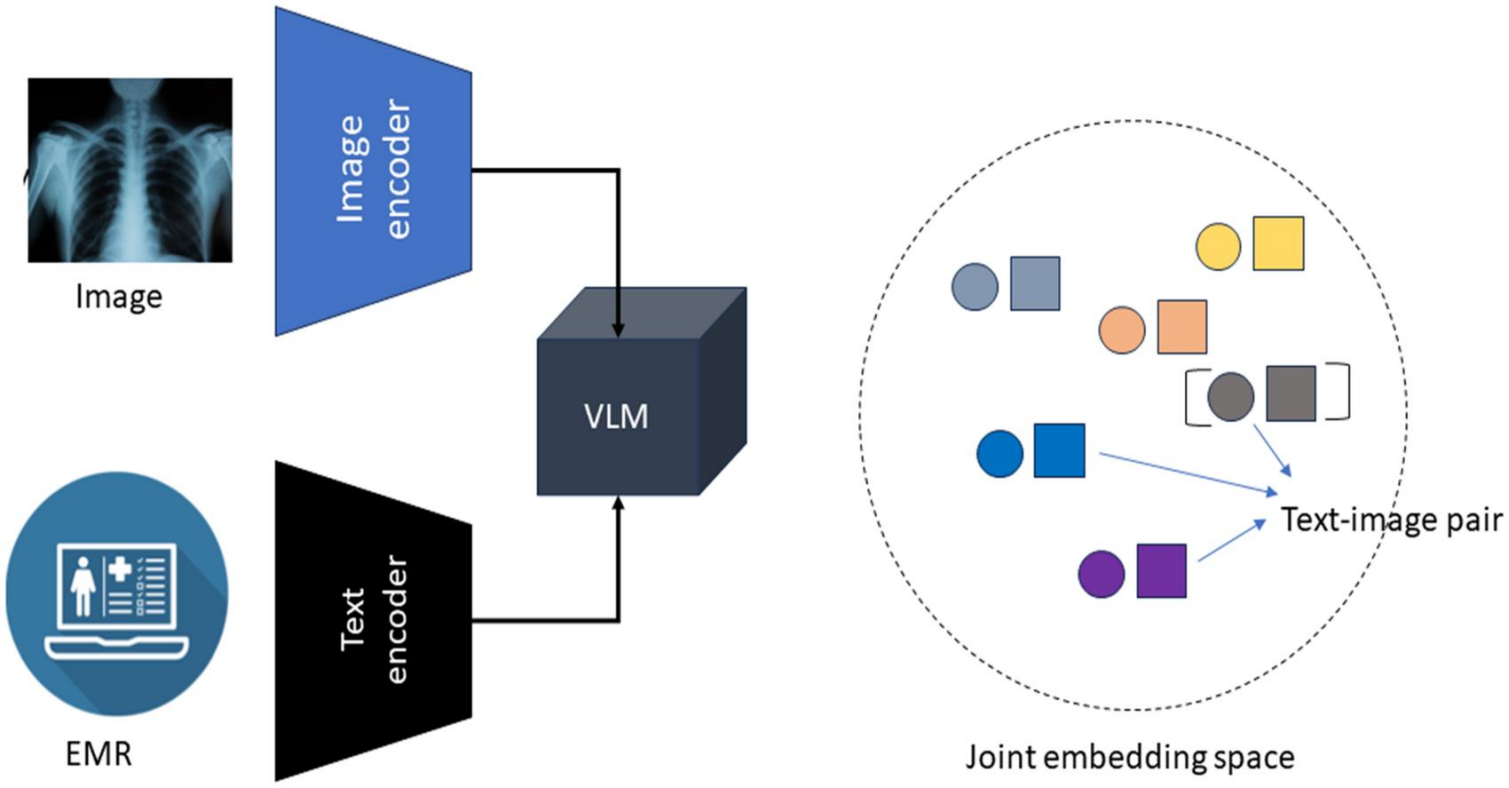
Joint embedding space generation using vision-language model (VLM)—image and text.

In recent years, large transformer models which are trained under self-supervision have revolutionized the fields of natural language and image processing. Large models (billions of trainable parameters) and training data set sizes (billions of word tokens) enable these models to show “emergent” behaviour, ie, they can perform task they were not explicitly trained for, ie, zero-shot performance. These models are known for their utility as zero-shot or few-shot learners for a wide variety of text-related tasks such as language generation and vision tasks such as image-to-image translation.^26–28^ Recently developed vision-language models (VLM) have shown surprisingly good performance on a variety of image and text-related downstream tasks like radiology report generation and visual question answering.^29–31^ Research in this field is rapidly moving towards multi-modal large models such as VLM that have joint embedding spaces to process visual and textual data to learn text-image pair interdependence (Figure 3). VLMs are generally composed of 2 encoders—one for each modality—and layers that jointly process both image and text features. MedCLIP and MedViLL are examples of joint encoder architectures.^29^^,^^30^ Transformer-based architectures have homogenized processes of both text and images as sequences of tokens (text) and image patches (images). For example, Vision and language transformer architecture (ViLT) uses 1 transformer-based encoder to encode an image-text pair.^32^ While MedCLIP was trained for the task of image-text matching using a contrastive loss, training of recent VLMs like ViLT included tasks like masked word prediction, image patch-word alignment, and image-text retrieval.

The primary challenge for training VLMs is that the inherently large size of these models requires huge training datasets—in the range of million text-image pairs. Examples of open-source datasets used to train these models are Public Multimodal Dataset (PMD)^33^ which contains about 70M image-text pairs, and several smaller datasets including Conceptual Captions,^34^ WIT,^35^ Localized Narratives,^36^ RedCaps,^37^ COCO.^38^ Developing annotated datasets at this scale in the healthcare domain is challenging which makes training VLMs for this domain difficult. In addition, benchmark datasets to robustly evaluate VLM performance are missing. Researchers are tackling this problem by combining several imaging and image-text datasets from clinical domains including Open-I,^39^ MIMIC-CXR,^40^ and VQA-RAD.^41^ MIMIC-CXR and VQA-RAD include image-text pairs from various imaging modalities (X-ray, CT, MR) and anatomical sites and have been used to train medical domain VLMs like MedViLL.^30^^,^^32^

As the availability of radiology datasets and computational resources improves, VLMs are increasingly being applied for several radiology tasks. The inherently self-supervised nature of VLM training where a model is optimized to learn correlation between image-text pairs, for example, radiology image and report pairs, is particularly suitable for radiology where the cost of manual annotation of images remains expensive. While VLMs were initially applied to 2-dimensional radiology images like chest X-ray,^29^ they are now being increasingly used for 3-dimensional radiology volumes like CT.^42^^,^^43^

### Ethical considerations/pitfalls of multimodal models in radiology

Multimodal models exhibit similar limitations to single modality models including limited explainability, poor generalizability when encountering out-of-distribution datasets (especially for traditional fusion models), and limited interpretability. Additional concerns arise when applying multimodal models. Review of several radiology and pathology models demonstrates that most of these models rely on clinical trial datasets which tend to be very structured and have a very strict exclusion criterion.^44^ This is because the radiology study must be matched to the pathology slide, and given varied appearance it’s impossible to generate overlays and image registrations. Therefore, it is unlikely that these types of models that are generated from clinical trial databases will demonstrate robustness when they encounter out-of-distribution data in a real-world setting.^44^ Another challenge of these models arises in the feature engineering which can inject human biases in what is considered important. Feature extraction pipelines, for example, with radiomics may lack robustness and standardization making it difficult to reproduce in datasets with variable acquisition parameters.^45^ Computational limitations may mandate to focus on the findings with high occurrences, for example, with radiogenomics—and this ignores the contribution of rare occurring cell types.

At baseline, deep learning models are challenging to interpret and explain due to the millions of model parameters. For multimodal models, traditional approaches may offer better explainability compared to VLMs. It is not clear if the large technical and data burden of multimodal data fusion outperforms single modality models or those that use simple regression approaches, and more research is necessary to fill this knowledge gap. The ability of deep learning models to encode “hidden characteristics” on images like self-reported race of patients is challenging as we combine different image types.^46^ Finally, a challenge of the use of multimodal models for developing biomarkers^47^ has shown that group differences are reported with demographic subgroups of race—a social and legal construct, that is captured differently across many care settings and is forbidden in some geographic regions. There is need to develop more subgroups from these multimodal data beyond Black and white patients—as there is a risk of strengthening historical biases that attempt to show different biological occurrences across different race groups.

## Conclusion

Large multimodal models like VLMs are pushing the boundaries of AI in radiology, but caution should be exercised to select the appropriate fusion approach for a task due to challenges of handling high-dimensional heterogeneous data in an end-to-end model. While existing pretrained VLMs are suitable for natural image tasks, careful finetuning may be required before their use in the healthcare domain. Graph-based fusion models present an alternative when limited training data are available and can also circumvent some of the challenges of missing data and model generalization. However, graph-based models have challenges with explainability. In cases where high-quality but limited multimodal data are available, traditional machine learning-based fusion models may be computationally efficient and explainable. Fusion models combining information from multiple sources are critical to delivering precision medicine by incorporating data from different modalities including imaging, text, tabular, and genomic sequences. As computational approaches advance, there will be several radiology toolkits available for harnessing multimodal data, as well as ethical pitfalls that must be addressed to ensure benefit for all patients. Table 2 summarises the pros and cons of various fusion strategies.

**Table 2.** Summary of pros and cons of various fusion strategies.

Approach	Pros	Cons
Traditional—early fusion	Explainable Ability to extract complimentary information in the various modalities	Must extract features from heterogeneous data (instead of using the raw data)—hence can be affected by the quality of feature extractor Inability to handle missing data Risk of overfitting due to high feature dimensionality Human-based feature curation and engineering can be biased and is time-intensive
Traditional—middle/joint fusion	Architectural innovation to support parallel extract features extractors. Backbone can be updated for better performance on the downstream task.	Inability to handle missing data Risk of overfitting due to high feature dimensionality Human-based feature curation and engineering can be biased and is time-intensive
Traditional—late fusion	Explainable No architectural innovation to extract features	Unable to learn complimentary information from different modalities Inability to handle missing data Human-based feature curation and engineering can be biased and is time-intensive
Graph neural networks	Minimal curation required Can handle missing data Robust generalizability to new datasets and missing data	Unstructured data may still need pretrained feature extractors Homogenization effect may occur when too many graph layers are included in the model Not explainable
Joint embedding of multimodal data	Require minimal dataset curation	Require large training datasets and compute No benchmark datasets for robust VLM performance evaluation
